# Supplementary figures and images for: Interruption of an MSH4 homolog blocks meiosis in metaphase I and eliminates spore formation in Pleurotus ostreatus
Source: PLoS One. 2020 Nov 4;15(11):e0241749. doi: 10.1371/journal.pone.0241749 (PMC7641404; doi:10.1371/journal.pone.0241749)

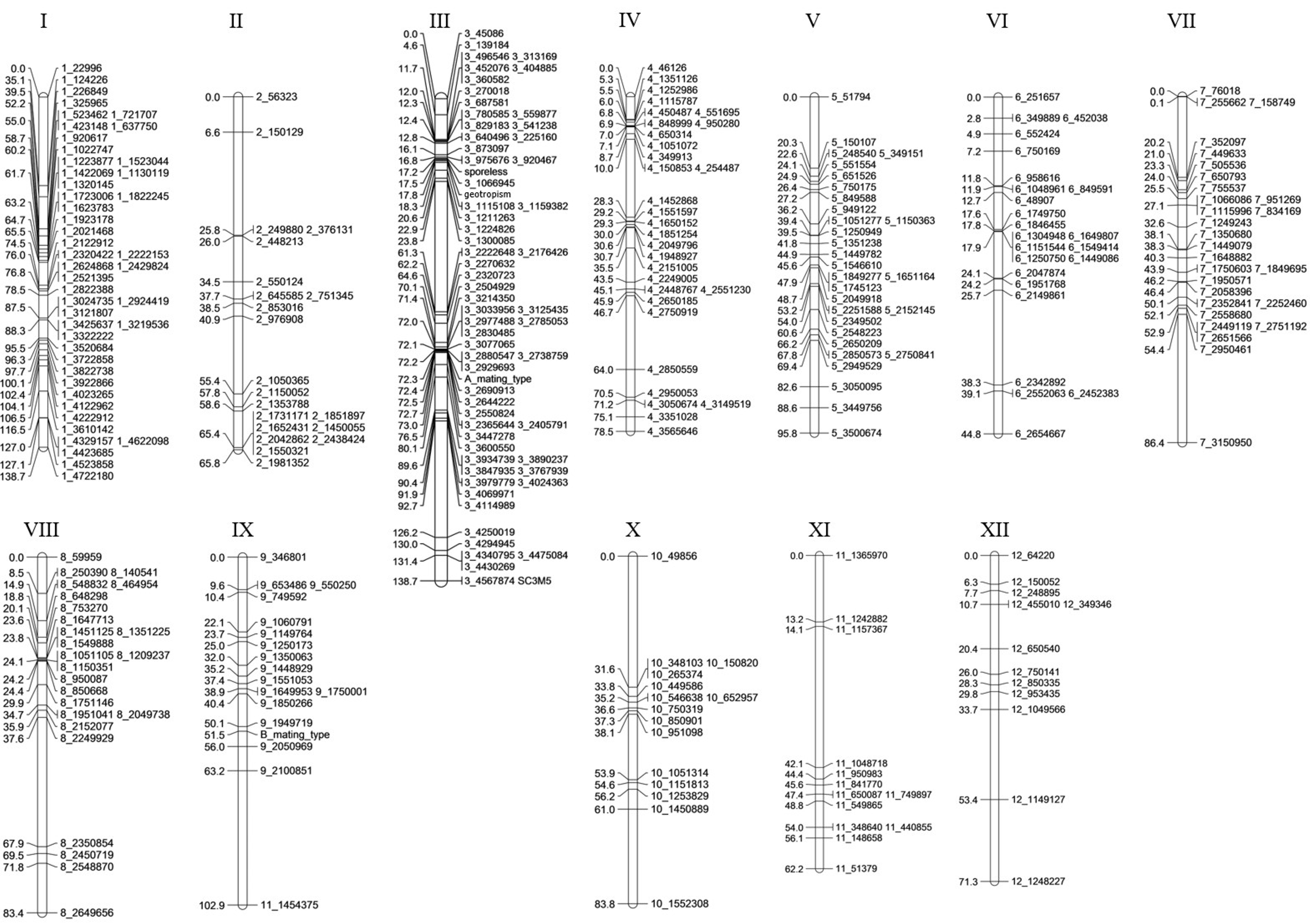

Supplement: S1 Fig — The linkage map is based on 188 monokaryotic progeny of the cross using 387 genetic markers and the phenotypes A mating-type, B mating-type, sporelessness and disturbed orientation of fruiting bodies (geotropism). (TIF) [file pone.0241749.s001.tif]

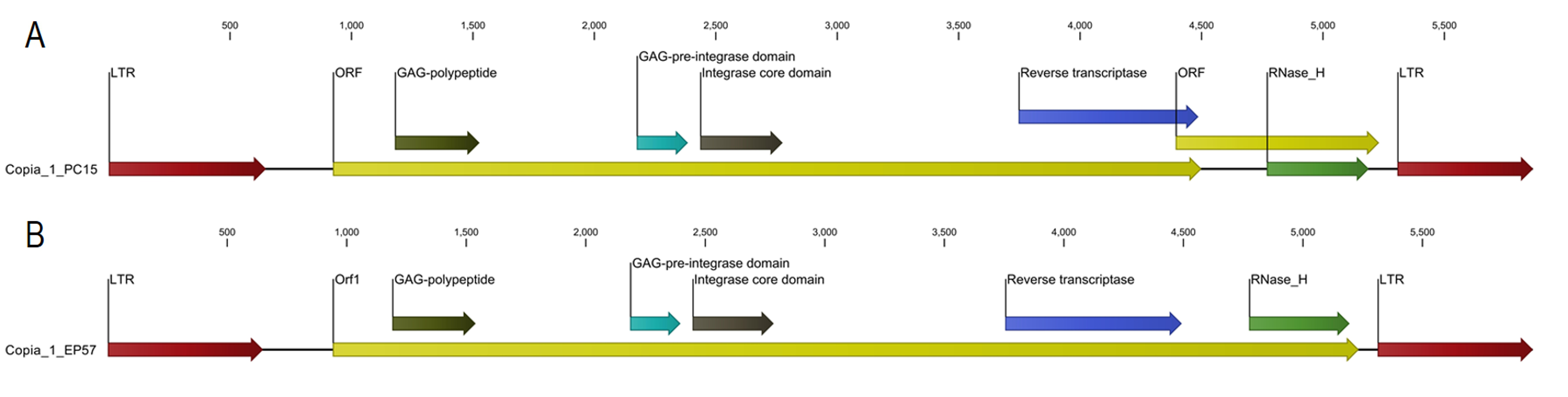

Supplement: S2 Fig — The retrotransposons located in the poMSH4 region of Sp+hap2 (A) and Sp-hap2 (B). (TIF) [file pone.0241749.s002.tif]

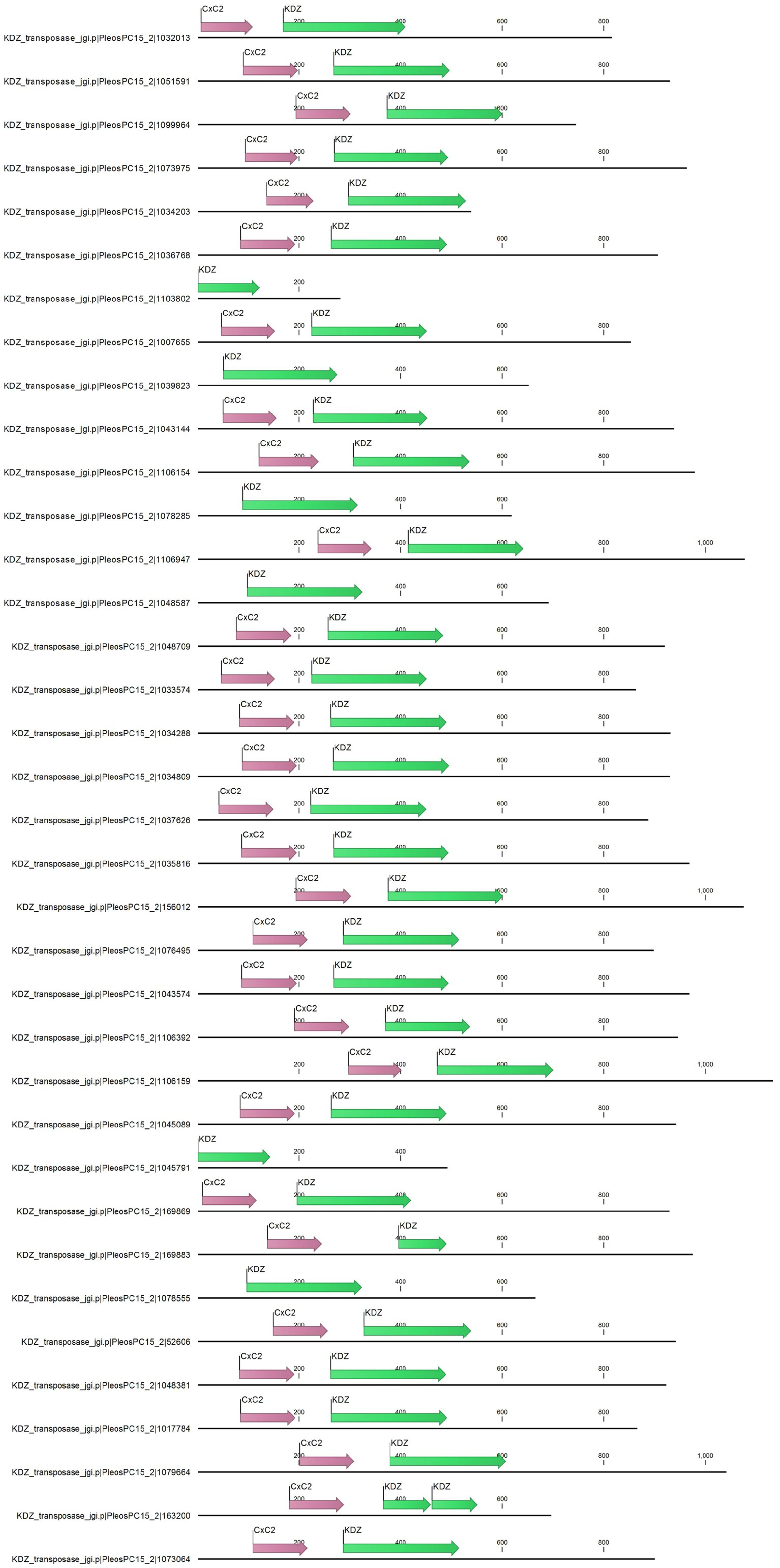

Supplement: S3 Fig — http://genome.jgi.doe.gov/PleosPC15_2. (TIF) [file pone.0241749.s003.tif]

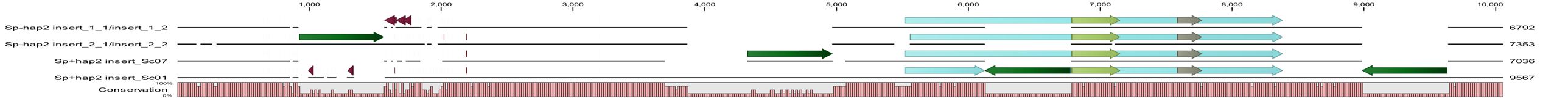

Supplement: S4 Fig — Insert_1_1 and insert_1_2 are identical copies, located in the poMSH4 region of Sp-hap2 (contig 00000008) of which insert_1_1 is integrated into poMSH4, disrupting the gene. Insert_2_1 and insert_2_2 are identical copies, located on contig 00003652 of Sp-hap2. Major difference between the “inserts” located in the poMSH4 region of Sp-hap2 and all the other copies of the “insert” is the absence of solo-LTRs of the Copia type RT (dark green). Next to that, there are some small differences in the number and composition of the small repeat units with the BC5NWY signature (Dark red). The light blue region represents the CxC5/CxC6 cysteine cluster encoding region with the CxC5 domain (light green) and the CxC6 domain (grey). (TIF) [file pone.0241749.s004.tif]
