# Supplementary material for: Interruption of an MSH4 homolog blocks meiosis in metaphase I and eliminates spore formation in Pleurotus ostreatus
Source: PLoS One. 2020 Nov 4;15(11):e0241749. doi: 10.1371/journal.pone.0241749 (PMC7641404; doi:10.1371/journal.pone.0241749)
Supplement: S5 Fig — Conserved domains indicated as described by Okuda et. al. [26]. The olive green boxes represent the ATP binding site and the stale blue represents the ABC transporter signature motif. Hash tags, upward-pointing arrows, downward-pointing arrows, plus signs and asterisks indicate the Walker A, Walker B, D-loop, Q-loop and H-loop respectively. (PDF) [file pone.0241749.s005.pdf]

```
poMSH4          MQASRPTTVSLSSFAYTTDHLQRFGTTSD---PTRLQSSRPQTGRPLTGRPQTG--RPQT
stpp1           MQTSRPTTMSLSSFAYTTDQPRRFGTTNDGSQSSRAQTSRPLTGRPQTGRPQTG--RPQT
scMSH4          MY-SRPNT----------------------GRPTTGASGRPQTG---TGRPWTGQTRPGT

poMSH4          GRPQTAVS--IRHEGSYVIALIEGRGVAREVGLAALDKDTGKVVLVQLADCQTYVKTLHQ
stpp1           GRPQTAVS--VRNEGNFVIALIEGRGVAREVGLAALDKDTGKVVLVQLADCQTYVKTLHQ
scMSH4          GRPGTAASNYVRHEASYIVAVLEGRGVAREVGMAALEKDTGQVVLVQISDCPTYVKTLHQ

poMSH4          MHLHTPSTILLPDTFISGAGPHGQPAPGA------SLLVEYIREEFPYVQLEPIGRKFWN
stpp1           MHLHTPSTILLPDTFISGAGPHGQPAQGA------SLLVEYIREEFPYVQLEPIGRKFWN
scMSH4          MHLHRPALVLVPDTFVASAD--GQTTSGGKRVNSTSVLVEAIEDEFPGVPVEAVGRKYWN

poMSH4          EAGGLEFIGQLCVDDDERTATMLAVSTKYYALSATCALFKYAEIKMNARFASRSLRIRYV
stpp1           EAGGLEFIGQLCVDDDERTATMLAVSTKYYALSATCALFKYAEIKMNARFASRSLRIRYV
scMSH4          ETSGLDFIQQLCVEDDERAGILLAVVNKYYTLSAASALFKHAELKLNIRFSSGSLRIRYA

poMSH4          PIEGTLMIDPDTVRNLELVASLTHKKSTHSLFGVLNHTYTAMAARLLRANILSPVTVQDA
stpp1           PVEGTLMIDPDTVRNLELVASLTHKKSTHSLFGVLNHTYTAMAARLLRANILSPVTVQDA
scMSH4          PVEGTMMIDPETARNLELVGNMTHKKSTHTLFGTLNHTFTAMGARLLRTNILSPITVLPS

poMSH4          INARLDFVEELICCEDKFTDIRDALKAFKKMDFDKLVASLAVSEARPVSTGKSAAARISH
stpp1           INARLDFVEELICCEDKFTDTRDALKTFKKIDFDKLVASLTVSEARPISTGKSAAARISQ
scMSH4          IDARLDVVEEFVKTEDRFNEVKDALRVLNKMDFDKLIASLAASEARPTNNAKPASTRVTQ

poMSH4          MLDLRTAIQSIPVLAQALSGGHCHLMQIMHHMLTDDRLAHIEGLVCSHLNDDTSLSKTGI
stpp1           MLDLRTAIQSIPVLAQALSGGRCHLLQILHHMLTDDRLAHIEGLVCSHLNDDTFLSKTGI
scMSH4          MLSLRNAVKTLPLLYKALEGSQSQLLKIIRDMLGDERLTKIQELVDERLNEDSTPSKGGI

poMSH4          AAVNARVYALKANKNHLLDVARETYKENVGDIYQLNRSLSEVHSLSLSLVYQE-TGFVFA
stpp1           AAVNARVYALKANKNHLLDVARETYKENVGDIYQLNRSLSEMHSLPLSLVYQE-TGFVFV
scMSH4          AAVNARVYAVKANCNRLLDVARETYRENIGDIFHLNRTLSDAHTLPLALVYQESTGFVFT

poMSH4          LKKDDLMGELPSGFINASIKRGSWYFSSMELKKMNARMKDALDETLLLSDRIIQELLDGI
stpp1           LKKDDLMDGLPSGFINASMKRGSWYFSSMELKKMNARMKDALDETLLLSDRIIQELLDGI
scMSH4          MKKDDLEGELPRGFLNVGLKKGKWVFTSMELKKMNARMKDALDETLLLSDKIIQDMVAEI

poMSH4          LEDVGVLYKASEAIALIDMLWSFTHFSIIHNCVRPEFTGTLAVKSGRHPILQLVQPAGSL
stpp1           LEDVGVLYKASEAIALIDMLWSFAHFSIIHNCVRPEFTGTLAVKSGRHPILQLVQPAGSV
scMSH4          IVDVGALYKASEAVALLDMLWSFAHASII----RPEFTGTLAIKSGKHPILETIQSAGSV
                                 ########                                 ++
poMSH4          VANDIYCDDSSHFQIIQGLNMSGKSTYLQQVGLLVIVALNGCFIPAEYGSFRVHDCLLSR
stpp1           VANDIYCDDSSHFQIIQGLNMSGKSTYLQQIGLLVIIALNGCFVPAEYGSFRVHDCLLSR
scMSH4          VPNDVYCDDSSCFQIVQGPNMSGKSTYLRQIALLLIVAMTGCFVPAEYASFRIHDALLTR

                ++                              ↑↑↑↑↑↑  ↓↓↓↓
poMSH4          LSNNDDMEKSLSTFGSEMATSTMILGMSTSQSLVLVDELGRGTSPQEGVGIAHAIAEGLI
stpp1           LSNNDDMEKSLSTFGSEMATSTMILGMSTPQSLVLVDELGRGTSPQEGVGIAHAIGEGLI
scMSH4          LSNDDDIERSLSTFASEMATSAMILGLATPKSLILLDELGRGTSPREGVGISHAIAEALV

                      *******
poMSH4          KQKCLTFFTTHFSELAQTLSKAPGVVNLHLSVQK--QPSSSFGLNFMYRITDGPLEKHEH
stpp1           KQKCFTFFTTHFSELAQTLSKASGIINLHLSVQK--QPLSSFGFNFMYRITDGPLEKHEH
scMSH4          RLKAFVFFATHFGELTTTLSRQPSVVNLHLSVQRSRRTTTNFGVTFQYRIVDGAPEDDSH

poMSH4          YGLQLAMLADFPKDLIDKGSQVAQNLDELYSQSSDHSSSSKIIAQRQALLRLQIQLKQAF
stpp1           YGLQLAMLADFPKDLIAKASQVAQSLDELYSRSSDYSSSSKIVAQRQTLVRLQIQLTQAF
scMSH4          YGLELARLADLPKDVLTEAKRVASQLAALHARHEESSESNKIAIRRKALLRLRTQLIQAY

poMSH4          QHSSLPQEELVEYLRRFQVHLAKLFVM--
stpp1           EHSSLPQEELVEYLKRFQTNLARLFVL--
scMSH4          EHSALPDQELLEYVARFQRDIAKAFVTVQ
```
