# Supplementary material for: Interruption of an MSH4 homolog blocks meiosis in metaphase I and eliminates spore formation in Pleurotus ostreatus
Source: PLoS One. 2020 Nov 4;15(11):e0241749. doi: 10.1371/journal.pone.0241749 (PMC7641404; doi:10.1371/journal.pone.0241749)
Supplement: S1 Table — Primers were designed to amplify genes including a 1 kb promoter and 500 bp terminator region using Pleurotus ostreatus Sp+hap2 as template. (DOCX) [file pone.0241749.s006.docx]

| Construct | Forward primer | Forward primer sequence | Reverse primer | Reverse primer sequence |
| --- | --- | --- | --- | --- |
| Sp. Reg. 02 | Sp_reg_F02 | caacgcagataagaaagcca | Sp_reg_R02 | aggggacgtagtagagga |
| Sp. Reg. 03 | Sp_reg_F03 | gtgtactccgctttggctt | Sp_reg_R03 | atcaccgccaacatcctca |
| Sp. Reg. 04 | Sp_reg_F04 | cttaaggacgcccgagttgttg | Sp_reg_R04 | tagatgagagtacggggagggg |
| Sp. Reg. 05 | Sp_reg_F05 | tgaccgctaggccgatcaaa | Sp_reg_R05 | agcgcgggcgtatatgggtt |
| Sp. Reg. 06 | Sp_reg_F06 | gaaggctgtgagtaatgggga | Sp_reg_R06 | ggtaggaggaagatgagggag |
| Sp. Reg. 07 | Sp_reg_F07 | cagaacgtaccgtcggaggctt | Sp_reg_R07 | aaacttgctaccagctccaggttc |
| Sp. Reg. 08 | Sp_reg_F08 | acggctaaataccttgacccaag | Sp_reg_R08 | tcgaaaacactcgcgccatc |
| Sp. Reg. 09 | Sp_reg_F09 | tcagatcatagcagcagtagcaaga | Sp_reg_R09 | ctgaacttttgtagccattcctcag |
| Sp. Reg. 12 | Sp_reg_F12 | cttctccgcctggatctcgt | Sp_reg_R12 | atggtactcgtgtcaacgcca |
| Sp. Reg. 13 | Sp_reg_F13 | actgggaagcgtcaacagcc | Sp_reg_R13 | gagccataccatagccaaggtg |
| Sp. Reg. 14 | Sp_reg_F14 | ttgcaggaagcgagcatgct | Sp_reg_R14 | acgaggaagcgtgctgaagc |
| MSH4 | MSH4_F | ggacggacggctaaataccttg | MSH4_R | ctctacacaaaccacacatgatgc |
