# Supplementary material for: Interruption of an MSH4 homolog blocks meiosis in metaphase I and eliminates spore formation in Pleurotus ostreatus
Source: PLoS One. 2020 Nov 4;15(11):e0241749. doi: 10.1371/journal.pone.0241749 (PMC7641404; doi:10.1371/journal.pone.0241749)
Supplement: S2 Table — (DOCX) [file pone.0241749.s007.docx]

| Transformant | Forward primer | Reverse primer |
| --- | --- | --- |
| ATCC58937 - Sp. Reg. 02 | FMSC3_F18 | FMSC3_R18 |
| ATCC58937 - Sp. Reg. 03 | FMSC3_F3 | FMSC3_R3 |
| ATCC58937 - Sp. Reg. 04 | FMSC3_F4 | FMSC3_R4 |
| ATCC58937 - Sp. Reg. 05 | FMSC3_F4 | FMSC3_R4 |
| ATCC58937 - Sp. Reg. 06 | FMSC3_F16 | FMSC3_R16 |
| ATCC58937 - Sp. Reg. 07 | FMSC3_F17 | FMSC3_R17 |
| ATCC58937 - Sp. Reg. 08 | FMSC3_F17 | FMSC3_R17 |
| ATCC58937 - Sp. Reg. 08 | FMSC3_F6 | FMSC3_R6 |
| ATCC58937 - Sp. Reg. 09 | FMSC3_F6 | FMSC3_R6 |
| ATCC58937 - Sp. Reg. 12 | FMSC3_F13 | FMSC3_R13 |
| ATCC58937 - Sp. Reg. 13 | FMSC3_F14 | FMSC3_R14 |
| ATCC58937 - Sp. Reg. 14 | FMSC3_F14 | FMSC3_R14 |
| ATCC58937 - MSH4 | FMSC3_F17 | FMSC3_R17 |
