# Supplementary material for: Interruption of an MSH4 homolog blocks meiosis in metaphase I and eliminates spore formation in Pleurotus ostreatus
Source: PLoS One. 2020 Nov 4;15(11):e0241749. doi: 10.1371/journal.pone.0241749 (PMC7641404; doi:10.1371/journal.pone.0241749)
Supplement: S3 Table — (DOCX) [file pone.0241749.s008.docx]

| Primer name | Forward primer |
| --- | --- |
| ∆msh4ufw | GGCCTAATAGGCCAATGTGGTGGCGGGAAG |
| ∆msh4urv | GGCCTCGCAGGCCTCTGGTGCAGCGTCTTC |
| ∆msh4dfw | GGCCTGCGAGGCCTACAAGGCGTCGGAAGC |
| ∆msh4drv | GGCCTATTAGGCCGCCGAATGGCGATCTTG |
| msh4ufscf | TGCAAAGGCGAGATAGTC |
| pdkufscr | TTGGTGACCTCCAGCCAGAG |
| pdkdfscf | GGCTGAGTCGTGGACTAAAG |
| msh4dfscr | AAGTACCCGCAGGAGAG |
