# Supplementary material for: Interruption of an MSH4 homolog blocks meiosis in metaphase I and eliminates spore formation in Pleurotus ostreatus
Source: PLoS One. 2020 Nov 4;15(11):e0241749. doi: 10.1371/journal.pone.0241749 (PMC7641404; doi:10.1371/journal.pone.0241749)
Supplement: Attachment — Submitted filename: Response to reviewers.docx [file pone.0241749.s015.docx]

PONE-D-20-21284
Interruption of an MSH4 homolog blocks meiosis in metaphase I and eliminates spore formation in Pleurotus ostreatus
PLOS ONE

Dear Dr Lustig,

Thank you for considering our manuscript for publication in PLOS ONE. The comments of the reviewer 1 and the Academic editor (AE) were addressed point by point below.

- The implicit argument that the MSH4 homolog is indeed MSH4 by the interchanging use of the terms "MSH4 homolog" and "MSH4" does not appear to be strong. Indeed, the 63% identity between related strains is large considering the relatively high conservation of MSH4. Is there any other information to back this up? In particular address the following three issues:
  1. Provide any evidence based on chromosomal synteny of MSH4 between S. commune and P. ostreatus. We apologize for the interchanging use of the terms MSH4 and MSH4 homolog. We meant to discuss the MSH4 homolog without directly claiming it has MSH4 activity. Based on the multiple sequence alignment and the BLAST results (see the following points 2 and 3) we are convinced that we can describe the studied gene as a MSH4 homolog and that additional synteny analysis, which only provides circumstantial evidence, is not needed.
  2. Provide information on whether the highly conserved structural regions of MSH4 are maintained between the two species?  In this regard, the sequence comparisons between MSH4 of S. commune and P. ostreatus must be directly compared in a Figure together with other known MSH4 genes from a couple of model organisms noting the highly conserved domains. Provide the additional coding details requested by Reviewer 1. A multiple sequence alignment (MSA) of the *poMSH4*, the *scMSH4* and the *P. pulmonarius stpp1* was added. Within this MSA the conserved domains as described by Okuda et. al., 2013 are indicated (S5 Fig). In the manuscript, line 442-444 is added: ‘and indicates a similar role of the encoded proteins in these organisms which is strengthened by the presence of similar structural domains in the encoded proteins (Suppl. Fig S5)’ referring to a supplemental file depicting the multiple sequence alignment.
  3. Present information regarding whether this MSH4 homolog is the only such homolog in P. ostreatus. Without further evidence, the authors should restrict the nomenclature to “MSH4 homolog” or state that “we will define the MSH4 homolog as poMSH4 for simplicity”. A BLAST search of the MSH4 sequence against the *P. ostreatus* PC15 reference sequence was performed. This resulted in only one hit. To the manuscript the following was added (line 271-272): ‘A BLAST search of the poMSH4 encoded sequence to Sp+hap2 revealed the presence of only one copy of this gene.’ We changed all *P.* ostreatus MSH4 or MSH4 homolog into “poMSH4” and for the *S. commune* MSH4 into “scMSH4”. This is indicated in the manuscript in line 38, line 90 and line 93.
- Address the fruiting body expression as requested by Reviewer 1.

Reviewer 1 requests addition of expression analysis (mRNA) of poMSH4 in different parts of the fruiting bodies. We feel that by showing that interruption of poMSH4 results in the sporeless trait and that reintroduction of the DNA encoding poMSH4 in the sporeless strain results in restoration of the phenotype is sufficient evidence that this gene plays a role in spore-formation. Furthermore, the goal of this study was mainly about finding the underlying gene(s) responsible for the sporeless trait. Further studies on the expression, regulation and mechanisms of the encoded protein are outside the scope of the manuscript and is a subject of further studies.

- Present the added detail in Figures S1 and S2 requested by Reviewer 1.

Reviewer 1 requests specification of the start codon and stop codon, promoter and terminator region in S1 and S2 file. We indicated the start codon, the mRNA and encoded sequence in file S1. As promoter and terminator regions 1 kb upstream and 0.5 kb downstream of the start and stop codon were taken, resp. (indicated in materials and methods).

Reviewer 1 mentioned that file S1 could not be opened. We do not know what is the problem, we can open it on our computers as a text file.

- Discuss the versality of the method more fully as described by Reviewer 1.

Reviewer 1 finds that the manuscript lacks data to claim its versatility. If the authors intend to claim its versatility in latter revising manuscript, please add the following data and descriptions:
・description of how to use MSH4 as a target in sporeless breeding. We ended our discussion with “MSH4 homologs might thus be a good candidate to generate also sporeless varieties in other edible basidiomycetes” To clarify and claim the versatility of the method we added the following (lines 476-482): “Since the obvious method to generate knockouts, i.e. CRISPR Cas9, is not an breeding method accepted by most consumers, mutants should be obtained in classical ways. Strains of these species containing a mutated MSH4 homolog may be obtained by screening a natural strain collection for MSH4 homolog mutants. In addition, the mutant may also be obtained by classical mutagenesis approaches followed by high-throughput screening of this mutant library as suggested before [26]. This marker can then be used for breeding, following a similar strategy as described for *P. ostreatus* [18, 19, 21] which resulted in a marketable sporeless strain.

・comparison between sequences (gene and amino acid) of MSH4, derived from P. ostreatus, S. commune and P. pulmonarius. Suppl. Fig S5 was added to the manuscript showing the a multiple sequence alignment of the *poMSH4*, the *scMSH4* and the *P. pulmonarius stpp1*. Conserved domains as described by Okuda et. al. 2013 were indicated. We added line 442-444; “and indicates a similar role of the encoded proteins in these organisms, which is strengthened by the presence of similar structural domains in the encoded proteins (Suppl. Fig S5).”

- Make the requested textual changes of Reviewer 1 and the AE :

1. Line 38: Change "MSH4" to "the meiotic recombination gene MSH4". Done
2. Line 41: Change "a MSH4 null mutant" to "the MSH4 null mutant in S. commune" if I understand the meaning properly. No this is the null mutant of P. ostreatus. We have clarified this by changing MSH4 to poMSH4 .
3. Line 43-45: Change ", and when......was observed" with , and the MSH4 null mutant confers an extremely low level of spore formation". Done
4. Line 45-46: Change "This confirms MSH4 as a" to "We propose that MSH4 is likely to be.." Done.

- Thank you for stating the following in the Financial Disclosure section:

'The authors received no specific funding for this work'

We note that one or more of the authors are employed by a commercial company: Ceradis B.V.

This is the current address of the Author Narges Sedaghat Telgerd, the work performed by this author was done when the author was employed by Wageningen University. Therefore, the funding statement does not need to be changed. The current address of this author was removed from the manuscript since this author is not the corresponding author.

a. Please provide an amended Funding Statement declaring this commercial affiliation, as well as a statement regarding the Role of Funders in your study. If the funding organization did not play a role in the study design, data collection and analysis, decision to publish, or preparation of the manuscript and only provided financial support in the form of authors' salaries and/or research materials, please review your statements relating to the author contributions, and ensure you have specifically and accurately indicated the role(s) that these authors had in your study. You can update author roles in the Author Contributions section of the online submission form. NA

Please also include the following statement within your amended Funding Statement.

“The funder provided support in the form of salaries for authors [insert relevant initials], but did not have any additional role in the study design, data collection and analysis, decision to publish, or preparation of the manuscript. The specific roles of these authors are articulated in the ‘author contributions’ section.” NA

If your commercial affiliation did play a role in your study, please state and explain this role within your updated Funding Statement. NA

 b. Please also provide an updated Competing Interests Statement declaring this commercial affiliation along with any other relevant declarations relating to employment, consultancy, patents, products in development, or marketed products, etc.  NA

Within your Competing Interests Statement, please confirm that this commercial affiliation does not alter your adherence to all PLOS ONE policies on sharing data and materials by including the following statement: "This does not alter our adherence to  PLOS ONE policies on sharing data and materials.” (as detailed online in our guide for authors [http://journals.plos.org/plosone/s/competing-interests](about:blank)) . If this adherence statement is not accurate and  there are restrictions on sharing of data and/or materials, please state these. Please note that we cannot proceed with consideration of your article until this information has been declared. NA

c. Please include both an updated Funding Statement and Competing Interests Statement in your cover letter. We will change the online submission form on your behalf. NA

- Please amend either the title on the online submission form (via Edit Submission) or the title in the manuscript so that they are identical. Upon resubmitting I will do this (there is a typo in the online submission).
- We note that you have included the phrase “data not shown” in your manuscript. Unfortunately, this does not meet our data sharing requirements. PLOS does not permit references to inaccessible data. We require that authors provide all relevant data within the paper, Supporting Information files, or in an acceptable, public repository. Please add a citation to support this phrase or upload the data that corresponds with these findings to a stable repository (such as Figshare or Dryad) and provide and URLs, DOIs, or accession numbers that may be used to access these data. Or, if the data are not a core part of the research being presented in your study, we ask that you remove the phrase that refers to these data.
  The phrase data not shown was removed and references to additional supplemental files S4, S5 and S6 were added (line 341-342). The supporting .gbk files S4, S5 and S6 contain the sequences of the region in Sp^-^hap2 containing the additional copies of the “insert” (S4), the “insert” homolog regions on scaffold 1 (S5) and scaffold 7 (S6) of Sp^+^hap2. In addition, we added the draft assembly Pleos_EP57v1.fasta as supplementary file S2. In the manuscript we refer three times to this sequence (line 287, 288 and 340).
- Further Review Comments to the Author

  Reviewer #1: The authors revealed a gene as causal agent of sporeless trait in P. ostreatus cultivar "SPOPPO". In addition, It is also interesting to clearly show the genetic relationship between sporeless and geotropism trait, which had been mentioned previously. However, this manuscript lacks basic data to their claims, which needs to be fulfilled for publishing.

  Major points:
  In this paper, the authors concluded that MSH4 is the causative gene of the sporeless trait in the mutants, and that MSH4 has versatility as a breeding target for developing sporeless cultivars in various species including Pleurotus sp. and Agaricales.

  1. Please consider adding the following data to ensure the reliability of data of MSH4 in P. ostreatus.
  ・List the accession number of MSH4 in P. ostreatus. The JGI accession number was added (line 267).
  ・Specify the start codon and stop codon, promoter and terminator region in S1 and S2 file. See answer to previous remarks of AE.
  ・Add the expression analysis by parts of fruiting bodies. See answer to previous remarks of AE.

  2. This manuscript lacks data to claim its versatility. If the authors intend to claim its versatility in latter revising manuscript, please add the following data and descriptions.
  ・description of how to use MSH4 as a target in sporeless breeding.
  ・comparison between sequences (gene and amino acid) of MSH4, derived from P. ostreatus, S. commune and P. pulmonarius. See answer to previous remarks of AE.

  Minor points:
  Line70-72
  Clarify the characteristics of ATCC58937, which is the base of SPOPPO.
  ・Degree of decrease in sporulation. The main goal of this study was to find the underlying gene(s) responsible for the sporeless trait of the SPOPPO strain. The strain ATCC58937 was the donor of the sporeless trait (100 % reduction of spores) in SPOPPO. Since sporelessness was mapped at the same location in both parental nuclei of ATCC58937, one of the parental homokaryons was selected to be used in this study. (100 % reduction of spores) was added to the manuscript (line 71-72).

・Specify the relationship between sporeless and geotropism, which has been mentioned in the past. Also indicate whether it is positive or negative geotropism.・About the mutation controlled by a single gene. Disruption of poMSH4 resulted in both the sporeless and disturbed orientation phenotype, reintroduction of the gene results in reversal of both phenotypes. Therefore, the relation we find between both phenotypes is the MSH4 gene. In theory we here deal with negative geotropism that is disturbed, the fruiting bodies normally grow slightly upwards (away from gravity) while the lamellae are oriented downwards. The indication ‘negative’ was added to the text (line 471) .

Line 321
Coprinopsis cinereus→Coprinopsis cinerea, Changed.

Line 457-458
What does the "cluster" mean? Effect by the geotropism? With “clusters” we mean “bunches”, and we changed the term in the manuscript.

Line473 substate→substrate? Changed.

Reviewer #2: Interruption of an MSH4 Homolog Blocks Meiosis in Metaphase I and Eliminates Spore Formation in Pleurotus ostreatus PONE-D-20-21284
The manuscript reports MSH4 gene as a breeding target for sporeless strains. The article is well written and present novelty to the state-of-the-art. The relevance of the advance reported is due to the poor development of breeding programs in cultivated mushrooms compared to other commercial crops such as plants. For instance, it sets the basis for the production of sporeless varieties such as the commonly Pleurotus variety SPOPPO widely cultivated in Europe. Sporeless varieties can prevent allergic sensitivity to airborne conidia, commonly in oyster mushroom in pickers. The genetic techniques employed are accurate and a protocol for Pleurotus transformation is presented. It is suitable for publication in Plos One in the form submitted.

Reviewer #1: Yasuhito Okuda

Reviewer #2**:** Jaime Carrasco
